# Model-based prediction of CD4 cells counts in HIV-infected adults on antiretroviral therapy in Northwest Ethiopia: A flexible mixed effects approach

**DOI:** 10.1371/journal.pone.0218514

**Published:** 2019-07-10

**Authors:** Tadesse Awoke Ayele, Alemayehu Worku, Yigzaw Kebede, Khangelani Zuma, Adetayo Kasim, Ziv Shkedy

**Affiliations:** 1 Epidemiology and Biostatistics, University of Gondar, Gondar, Ethiopia; 2 School of Public Health, Addis Ababa University, Addis Ababa, Ethiopia; 3 Health Sciences Research Council, Pretoria, South Africa; 4 Wolfson Research Institute, Durham University, Durham, United Kingdom; 5 I-BioStat, Hasselt University, Diepenbeek, Belgium; University of Pittsburgh Centre for Vaccine Research, UNITED STATES

## Abstract

**Background:**

CD4 cell counts is widely used as a biomarker for treatment progression when studying the efficacy of drugs to treat HIV-infected patients. In the past, it had been also used in determining eligibility to initiate antiretroviral therapy. The main aim of this was to model the evolution of CD4 counts over time and use this model for an early prediction of subject-specific time to cross a pre-specified CD4 threshold.

**Methods:**

Hospital based retrospective cohort study of HIV-infected patients was conducted from January 2009 to December 2014 at University of Gondar hospital, Northwest Ethiopia. Fractional polynomial random effect model is used to model the evolution of CD4 counts over time in response to treatment and to estimate the individual probability to be above a pre-selected CD4 threshold. Human subject research approval for this study was received from University of Gondar Research Ethics Committee and the medical director of the hospital.

**Results:**

A total of 1347 patients were included in the analysis presented in this paper. The cohort contributed a total of 236.58 per 100 person-years of follow-up. Later the data were divided into two periods: the first is the estimation period in which the parameters of the model are estimated and the second is the prediction period. Based on the parameters from the estimation period, model based prediction for the time to cross a threshold was estimated. The correlations between observed and predicted values of CD4 levels in the estimation period were 0.977 and 0.982 for Neverapine and Efavirenz containing regimens, respectively; while the correlation between the observed and predicted CD4 counts in the prediction period are 0.742 and 0.805 for NVP and EFV, respectively.

**Conclusions:**

The model enables us to estimate a subject-specific expected time to cross a CD4 threshold and to estimate a subject-specific probability to have CD4 count above a pre-specified threshold at each time point. By predicting long-term outcomes of CD4 count of the patients one can advise patient about the potential ART benefits that accrue in the long-term.

## Introduction

Human Immunodeficiency Virus (HIV) destroys CD4 cell count, which result to an increased plasma HIV RNA levels and experience an Acquired Immune Deficiency Syndrome (AIDS) in the long-run [1, 2]. Globally, a total of 36.7 million people are living with HIV [3] from whom two-thirds are from low and middle-income countries. Sub-Saharan Africa carries the highest burden of the diseases, 71% of the global total, but only about 12% of the world’s population [4].

Despite the drawbacks in the development of a successful vaccine against HIV, the development of therapeutic regimens using drug combinations has significantly increased survival and reduced HIV-associated morbidities in HIV-infected individuals [5, 6]. Countries use World Health Organization (WHO) guideline to roll-out antiretroviral therapy (ART). CD4 cell counts was used to initiate standard first line regimens and to switch to second line during the previous time [7].

The primary goal of ART is to reduce HIV-related morbidity and mortality, prolong survival, improve the quality of life, restore and preserve immunologic function and prevent HIV-transmission [8]. The level of CD4 cell counts is routinely used for monitoring response to ART in HIV-infected patients. According to the CD4 cell count criteria, a patient would be eligible when his/her CD4 cells counts dropped below a given threshold value. The threshold value has been changed from less than 200 *cells*/*mm*^3^ in 2006 to less than 350 *cells*/*mm*^3^ in 2010 [9, 10]. The WHO 2013 guideline recommend that ART be initiated for all patients with CD4 count 500 *cells*/*mm*^3^ or less [11]. In 2015, WHO recommended HIV-treat all approach [12] based on two clinical trial outcomes [13, 14]. However, several studies are against earlier initiation of ART in patients who have high CD4 cell counts [15, 16]. This is because early exposure to ART may precipitate early evolution of resistance and unnecessary side-effects [17]. The standard therapy consists of three nucleoside reverse transcriptase inhibitors (NRTIs): Zidovudine (AZT), Tenofovir (TDF), and Stavudine (D4T) and one non-nucleoside reverse transcriptase inhibitor (NNRTI): nevirapine (NVP) or efavirenz (EFV) [18].

Limited studies were focused on modeling the CD4 cell counts trend over time for patients on ART especially in Sub-Saharan Africa. A study in Northwest Ethiopia [19] used semi-parametric mixed effect model to investigate CD4 counts response to treatments. Similarly, a study in eastern Ethiopia [20] used a linear mixed model which ignores the non-linear nature of the evolution. Another longitudinal study in Uganda [21] included cubic time effect to account the non-linear nature of CD4 count. In the current study, we proposed a flexible parametric modeling, the fractional polynomials framework [22–26], to predict a subject specific evolution of CD4 counts over time. We focus on two main issues: (1) an early prediction of CD4 counts under a specific treatment and (2) the estimation of the time to cross a given CD4 threshold under treatment. The later allows us to compare the efficacy between different treatments.

This paper is structured as follows; Brief introduction is presented in the first part. In the second section, we describe the data and the proposed modeling approached is formulated. The proposed model is applied to the data and the results are reported in third section. A discussion is provided in the forth section.

## Methods

### Ethical clearance

A human subject research approval for this study was received from Institutional Review Board (IRB) of the University of Gondar. As the study was retrospective, the IRB waived that the research could be done based on record review without contacting the patients. Support letter was obtained from the medical director office of the hospital for retrieving retrospective data from the database and records. All the information was kept confidential, and no individual identifiers were collected.

### Data

**Fig 1:**
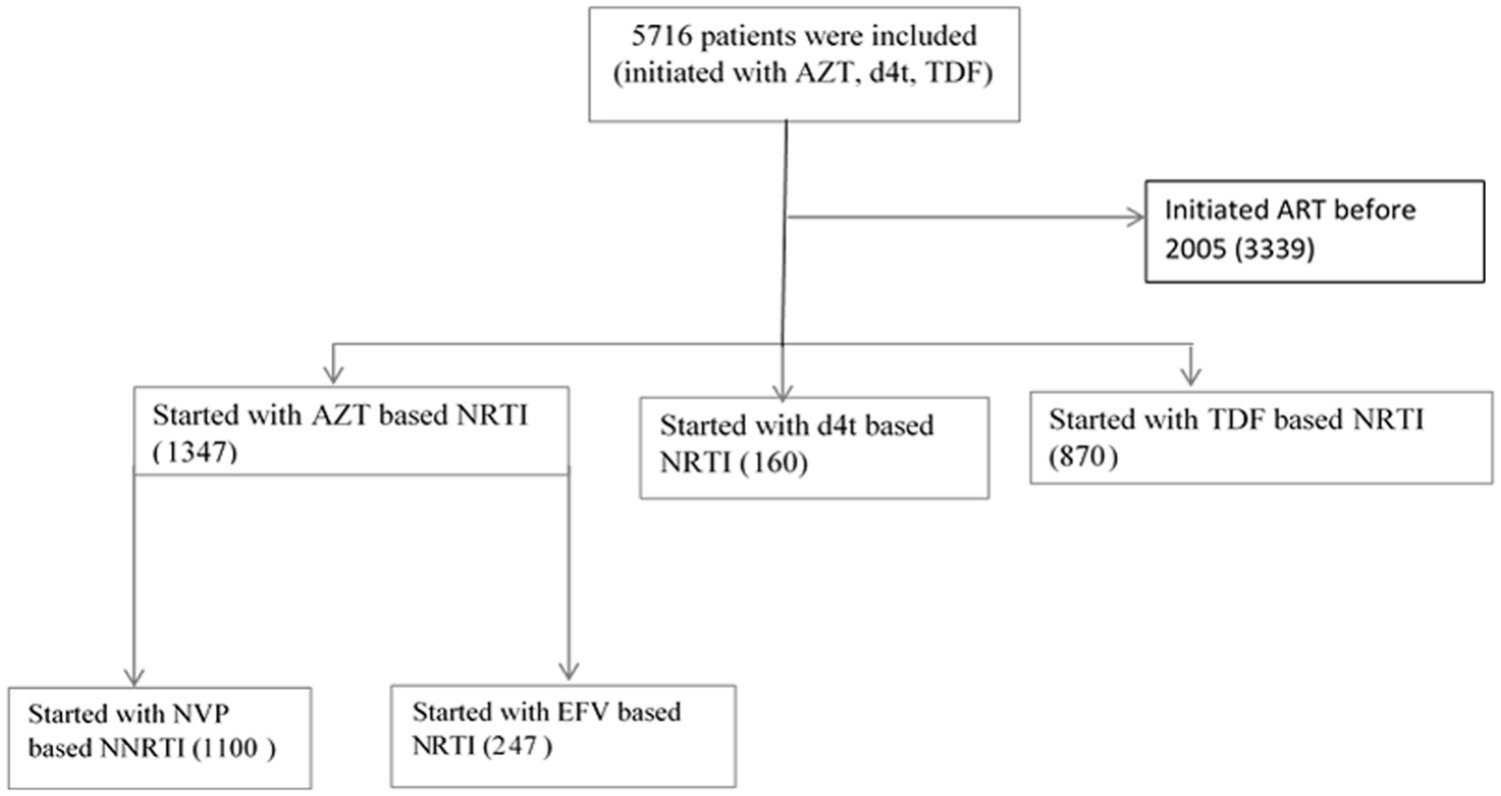
An illustration for the data flow diagram; A total of 5716 patients included, from which 58.4% initiated before 2005. A total of 1347 patients were included for final analysis (1100 NVP group and 247 EFV group).

The data were collected from tertiary referral hospital in Northwest Ethiopia. The hospital serves a population of about 5 million people in the region and neighbours. The hospital has a voluntary counseling and testing clinic where both self-referred individuals and physician referred patients are tested for HIV. A total of 14, 000 HIV-infected individuals ever visited the ART clinic in the hospital, of whom 8927 ever started ART. Free ART service is offered to the population since 2005. The inclusion criteria for the study presented in this paper were (1): having at least two visits and one CD4 cell count measurements, (2) initiated ART within the study period, (3) initiated with either NVP or EFV as NNRTIs treatment groups, and (4) initiated with AZT NRTI backbones. In total, 2550 adults HIV/AIDS patients met the criteria, of whom 1347 initiated with AZT containing regimen. Available case analysis was used to handle missing data. The flow diagram of the data is presented in Fig 1.

### Modeling CD4 cell counts using subject specific models

We considered a linear mixed effects model [27] given by

$$\begin{array}{c} Y_{it_{ij}}=X_{i}\beta +Z_{i}b_{i}+\varepsilon_{it_{ij}}. \end{array}$$ (1)

Here, $Y_{it_{ij}}(i=1,\ldots ,n_{i},j=1,\ldots ,m_{i}$) is *n*_*i*_-dimensional response vector of log transformed CD4 counts of the *i*^*th*^ individual at time *t*_*ij*_, **X**_*i*_ is *n*_*i*_ × *p* and *n*_*i*_ × *q* dimensional fixed effect model matrices, whereas **Z**_*i*_ is a random effects model matrices. The covariates Gender, WHO stage, age and NNRTI treatment were treated as fixed effect covariates and time (in month) is the random effect covariate. Note that *t*_*ij*_ is not the same for all patients which follow different schedule for their visits. The vector ***β*** is a *p*-dimensional vector of fixed effects and **b**_*i*_ is a *q*-dimensional subject specific vector of random effects $b_{i}∼N\left(0,D\right)$ and $\varepsilon_{it_{ij}}$ is the random error term, $\varepsilon_{it_{ij}}∼N\left(0,\sigma^{2}I_{n_{i}}\right)$. In what follows we discuss the usage of the mixed effects model as a tool to obtain a model based prediction of the CD4 counts, and the estimation of the time to cross a CD4 count threshold under a given treatment regimen. According to individual and average evolution, CD4 cell count was non-linear over time. Thus, a flexible model formulation for the mean structure using fractional polynomial random effect model is presented.

### Model based prediction

The primary goal of the analysis presented in this paper is to obtained a model based subject specific prediction under a specific NNRTI treatment regimen of long term level of CD4 counts as early as possible. For this purpose, a two stage procedure was used. First, a mixed effect model is fitted using the data between 0 to 30 months. We term this period as *the estimation period*. In the second stage, the fitted model is used to predict the CD4 counts in the period of 31 to 68 months. We term this period as *the prediction period*. We determine the period for estimation and prediction by considering the length of the study period. Initially a period of 30 months of follow up was used as the period of prediction and analysis was conducted. Later sensitivity analysis was performed and the analysis supported the initial decision on the period of estimation.

**Fig 2:**
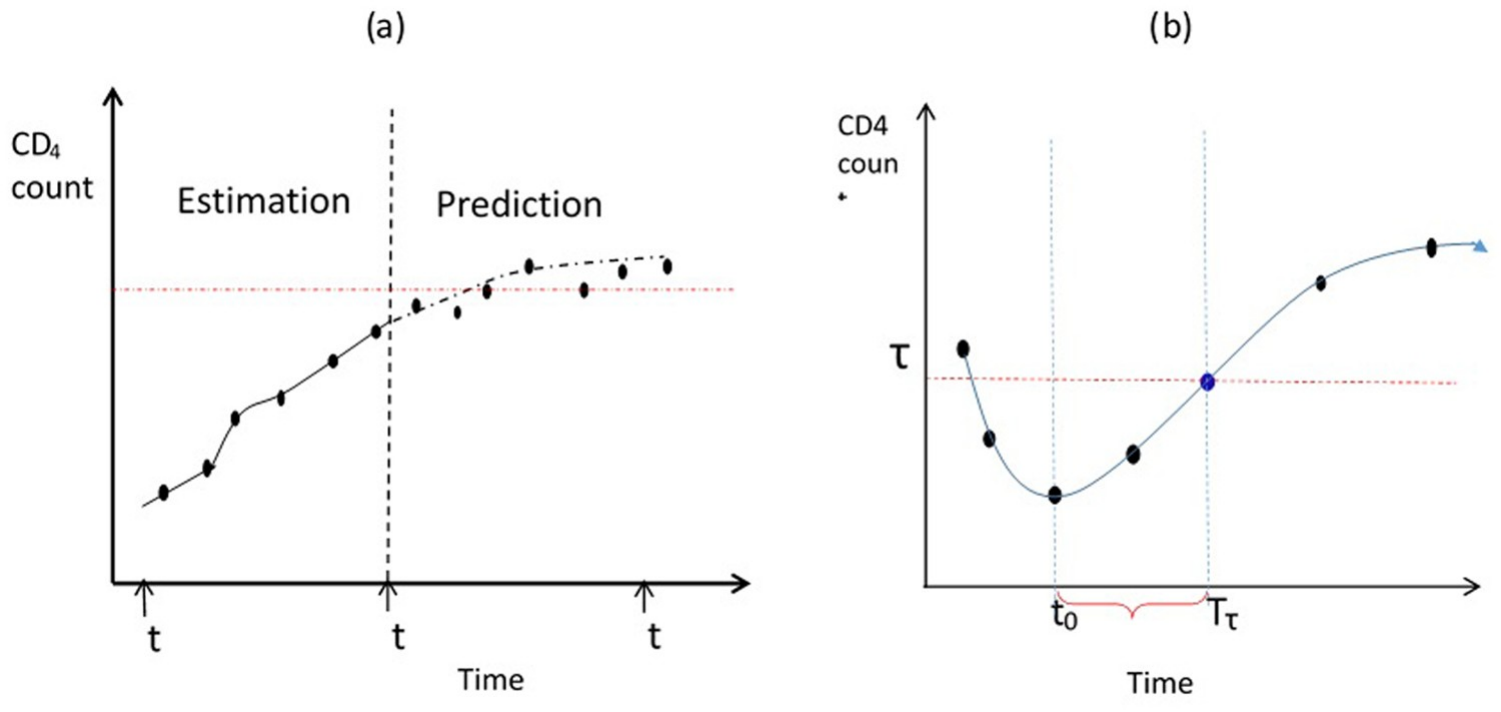
An illustration for model based prediction. Panel a: subject specific CD4 counts are divided into the estimation and prediction period. Panel b: an illustration of the time to cross a pre-specified threshold.

**Fig 3:**
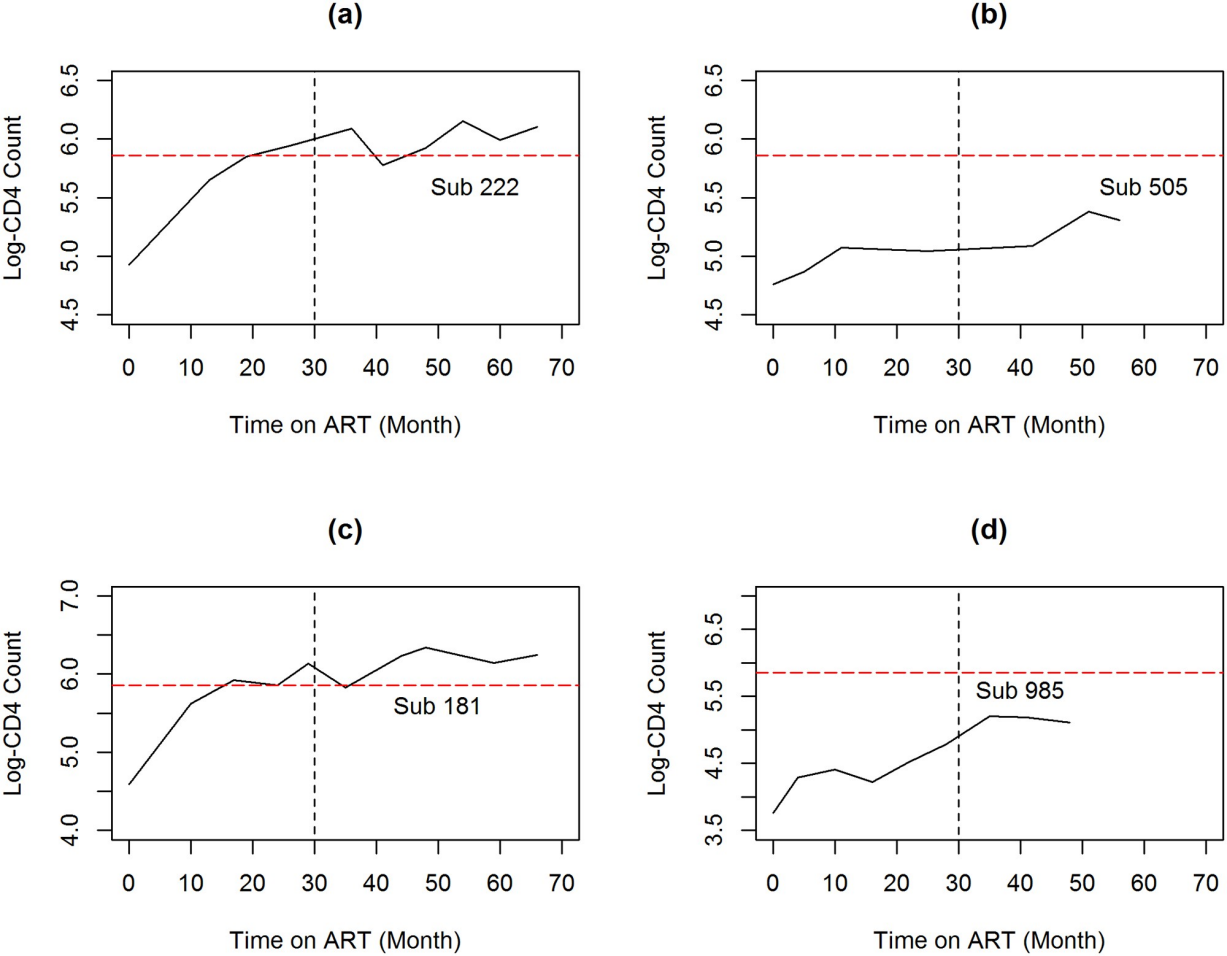
Individual profiles for patients who initiated ART with NVP (top panel) and EFV (bottom panel) containing regimens who cross and remain below the threshold.

The observed and predicted values in both estimation and prediction period are compared and their correlation was calculated in order to determine how good the model predicts the long-term CD4 counts. The procedure is illustrated in Fig 2a, where *t*_0_, *t*_*i*_, and *t*_*k*_ represented the initiation time of ART treatment, estimation period and prediction period, respectively. Fig 3 illustrate profile for selected individuals.

### Model based prediction of time to cross a pre-specified CD4 threshold

The linear mixed effect model formulated in Eq 1 can be used to predict the time that a subject will cross a pre-specified threshold level of CD4 counts. Let *τ* be a thresholds value, *t*_0_ is the time to ART initiation, and ⊤_*τ*_ the first time in which the subject CD4 counts crossed *τ* defined by

$$\begin{array}{c} {⊤}_{\tau }=min\left\{j\geq 1:Y_{it_{ij}}\geq \tau \right\}, \end{array}$$ (2)

Fig 2b illustrates schematically the trajectory of an individual who initiated ART at *t*_0_. In practice, three different threshold values of CD4 level were used for illustration: *τ* = *ln*(200) = 5.2983, *τ* = *ln*(350) = 5.8579, and *τ* = *ln*(500) = 6.21461 log(*cells*/*mm*^3^). Natural logarithm transformation was used. The threshold value of 200 *cells*/*mm*^3^ was recommended by WHO 2006 [9] as a criteria to initiate ART. In 2010, the criteria was modified to 350 *cells*/*mm*^3^ [10] and increased the threshold to 500 *cells*/*mm*^3^ in 2013 [11]. Since 2014, test and treat all approach is used irrespective of CD4 count level [12]. The current recommendation is viral load in order to monitor the progression of the virus and Ethiopia started implementing this recommendation in 2016 [28].

Based on the results presented in Reddy et al [29] the expected time for the *i*^*th*^ individual to reach a CD4 count greater or equal to the threshold *τ* can be express as

$$\begin{array}{c} \begin{array}{cc} E({⊤}_{\tau }) & =t_{i1}P\left(Y_{it_{i1}}\geq \tau \right)+t_{i2}P\left(Y_{it_{i1}}<\tau ,Y_{it_{i2}}\geq \tau \right)\\ & +t_{i3}P\left(Y_{it_{i1}}<\tau ,Y_{it_{i2}}<\tau ,Y_{it_{i3}}\geq \tau \right)+\ldots \\ & =\sum_{j=1}^{\infty }t_{ij}S_{ij}. \end{array} \end{array}$$ (3)

Here *t*_*ij*_ is the time corresponding to the *j*^*th*^ visit for the *i*^*th*^ individual, and *S*_*ij*_ is the probability of the *i*^*th*^ individual experiencing the event or stopping at *t*_*ij*_. In practice, the infinite series will be truncated at a time point relevant to the study subjects. Condition on the random effects, the mixed model formulated in Eq 1 implies the so called conditional model [27], that is

$$\begin{array}{c} Y_{ij}|b_{i}∼\text{N}(X_{i}\beta +Z_{i}b_{i},\Sigma_{i}). \end{array}$$

Hence, the joint probability that form *S*_*ij*_ reduces to the product of the individual probabilities, which can be expressed as

$$\begin{array}{c} \begin{array}{cc} S_{ij}\left(X_{i},Z_{i},b_{i},\beta \right) & =P\left(Y_{it_{i1}}<\tau \right)P\left(Y_{it_{i2}}<\tau \right)P\left(Y_{it_{i3}}<\tau \right),\ldots ,P\left(Y_{it_{ij}}\geq \tau \right)\\ & =\left[{\tilde{\phi }}_{i1}\left(\tau \right)\right]\left[{\tilde{\phi }}_{i2}\left(\tau \right)\right],\ldots ,\left[{\tilde{\phi }}_{ij-1}\right]\left[1-{\tilde{\phi }}_{ij}\left(\tau \right)\right], \end{array} \end{array}$$ (4)

where ${\tilde{\phi }}_{ij}\left(\tau \right)$ is a cumulative normal distribution with mean *X*_*i*_***β*** + *Z*_*i*_***b***_*i*_ and variance *σ*^2^, that is

$$\begin{array}{c} \begin{array}{c} {\tilde{\phi }}_{ij}\left(\tau \right)=\phi (\frac{\tau -X_{i}\beta -Z_{i}b_{i}}{\sigma }), \end{array} \end{array}$$ (5)

Note that both fixed and random effects defined in the mixed model formulated in Eq 1 are used to calculate ${\tilde{\phi }}_{ij}\left(\tau \right)$. We elaborate this point in the next section.

As shown by Reddy et al [29], the non-parametric bootstrap method can be used to compute standard errors and 95% confidence intervals for ${\hat{⊤}}_{\tau }$. Four steps procedure is applied to compute the standard errors and confidence intervals:

1. Individual *i* is removed from the full dataset resulting *N* − 1 samples.
2. Sample *N* − 1 subjects with replacement from the dataset in step 1.
3. Append the data of individual *i* to the bootstrap sample.
4. compute ${\hat{⊤}}_{\tau }$.

This procedure is repeated 1000 times.

### Flexible modeling of the mean structure

The fractional polynomial model was proposed by Royston and Altman [22] as a flexible parametric approach to describe the dependency between a response of primary interest and continuous covariates. The responses of primary interest in the current application is the log transformed CD4 cell counts and the covariate is time under ART treatment measured in months. The mean structure of an *m* order fractional polynomial model can be formulated as

$$\begin{array}{c} \sum_{l=0}^{m}\beta_{l}H_{l}\left(t\right)+\sum_{l=0}^{m}b_{li}H_{l}\left(t\right), \end{array}$$ (6)

where *m* is an integer, *p*_1_ ≤ *p*_2_ ≤ ⋯ ≤ *p*_*m*_ is a sequence of known powers and *H*_*l*_(*t*) is a transformation function given by

$$\begin{array}{c} H_{l}\left(t\right)=\{\begin{array}{cc} t^{p_{l}} & \text{if}\;p_{l}\neq p_{l-1}\;,\\ H_{l-1}\left(t\right)\times \log\left(t\right) & \text{if}\;p_{l}=p_{l-1}, \end{array} \end{array}$$ (7)

with *p*_0_ = 0 and *H*_0_(*t*) = 1. Note that there are two components in the mean structure. The first consists of the fixed parameters *βl* and the later the subject specific parameters *b*_*li*_.

For the analysis presented in this paper, both first (*m* = 1) and second (*m* = 2) order factional polynomials mixed effect models were considered and compared. To select the power of the model, powers in the range {−2, −1.5, …, 2.5, 3} were considered. Akaike Information Criteria (AIC, [30]) was used to choose the appropriate model. For a second order mixed effect fractional polynomial the mean structure is given by

$$\begin{array}{c} f\left(t_{ij}\right)=\left(\beta_{0}+b_{0i}\right)+\left(\beta_{1}+b_{1i}\right)t_{ij}^{p_{1}}+\left(\beta_{2}+b_{2i}\right)t_{ij}^{p_{2}}, \end{array}$$ (8)

Here, *β*_0_, *β*_1_, and *β*_2_ are fixed effect parameters, and *b*_0*i*_, *b*_1*i*_, and *b*_2*i*_ are subject specific parameters. To compute cumulative probability above the threshold, the unknown quantities of Eq 5 were substituted by their estimate; $X_{i}\hat{\beta }={\hat{\beta }}_{0}+{\hat{\beta }}_{1}t_{ij}^{p_{1}}+{\hat{\beta }}_{2}t_{ij}^{p_{2}}$ and $Z_{i}{\hat{b}}_{i}={\hat{b}}_{0i}+{\hat{b}}_{1i}t_{ij}^{p_{1}}+{\hat{b}}_{2i}t_{ij}^{p_{2}}$. R packages were used to perform all statistical analysis.

## Results

### Descriptive analysis

The median follow up period is 27.10 months (IQR = 12.10-43.10). The cohort contributed a total of 236.58 per 100 person years of follow up. The contributions were 237.11 per 100 person years and 234.28 per 100 person years by NVP and EFV containing regimens, respectively. The difference between the two treatment groups with regard to time contributed was 2.83 person years. The median number of repeated measurements was 3 (IQR = 2-6) with a maximum of 11 measurements per patient. At baseline, the absolute CD4 cell counts ranges between 2 and 2057 cells/*mm*^3^. To describe the pattern of CD4 change, different types of transformations are used [31]. In this study, log transformed CD4 cell count was used.

**Fig 4:**
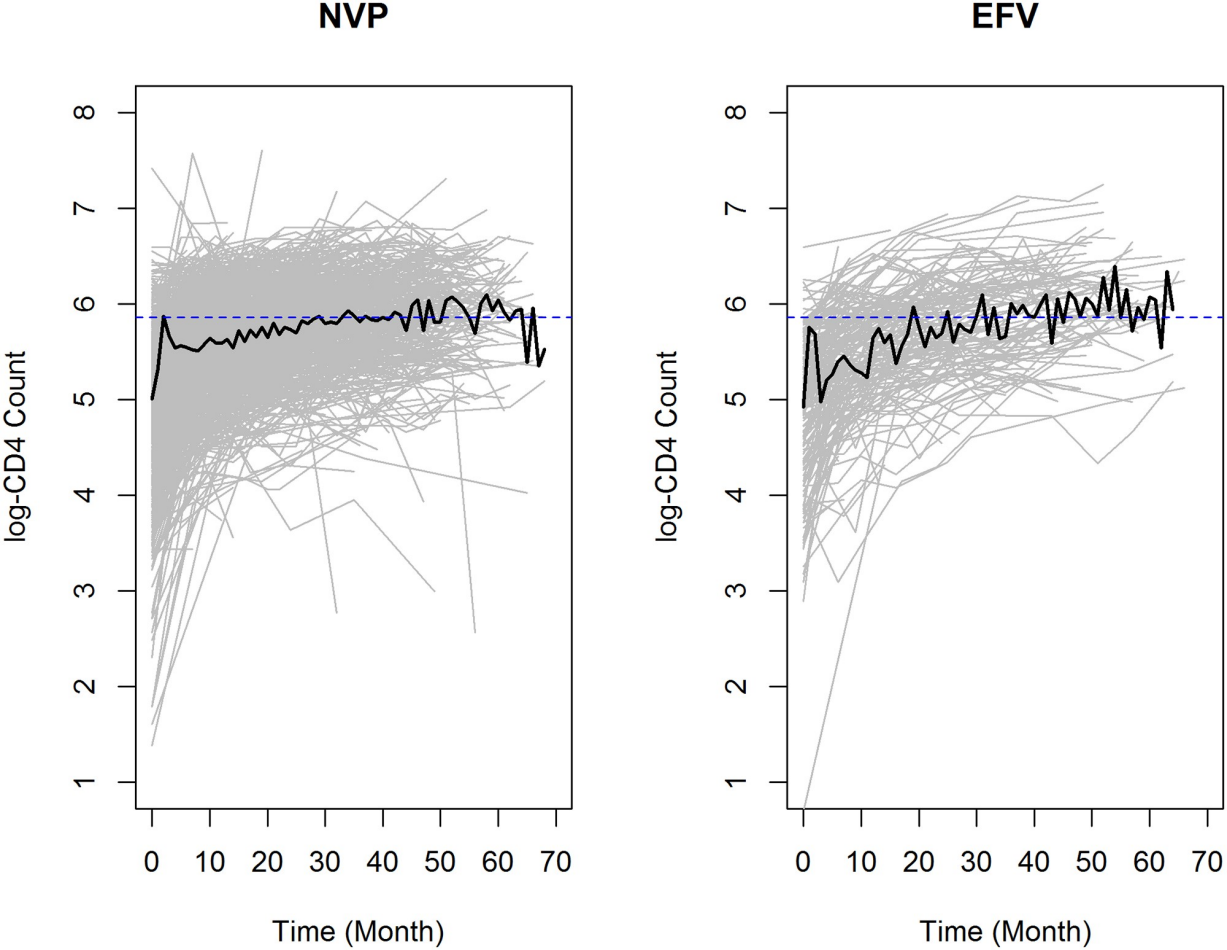
Individual and average profiles for patients who initiated ART with NVP (left panel) and EFV (right panel) containing regimens.

The evolution of CD4 cell counts over time for both treatment groups is shown in Fig 4 and reveal substantial variability between subjects. At baseline, 68.09% and 64.32% of the patients had CD4 cell counts below 200 *cells*/*mm*^3^ who initiated with EFV and NVP containing regimens, respectively. The percentage of patients who has CD4 cell count lower than 350 *cells*/*mm*^3^ increased to 94.53% and 91.97% for those who initiated with EFV and NVP containing regimen, respectively.

### Model based prediction of CD4 cell counts: Estimation period 0-68 months

According to the AIC value, a second order fractional polynomial mixed effect model (FP2) was selected. The smallest value of AIC was obtained at (*p*_1_ = 0, *p*_2_ = 0.5) and (*p*_1_ = 0, *p*_2_ = 0) for NVP and EFV containing ART regimen, respectively (S1 and S2 Figs).

**Fig 5:**
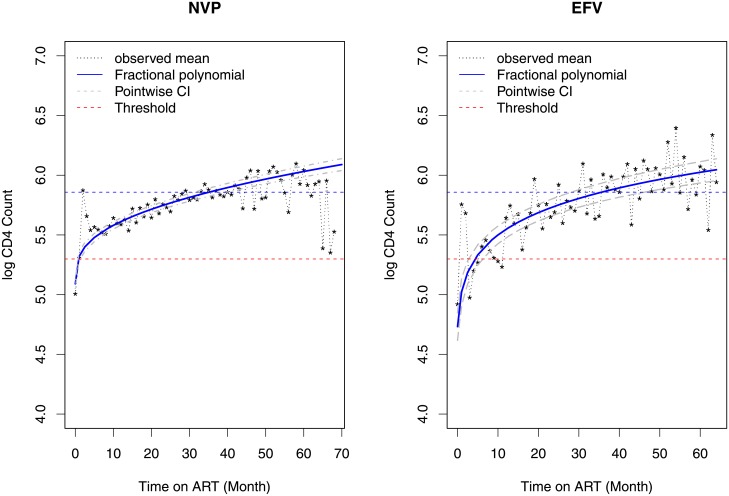
Model based predicted means for log(CD4) counts for NVP (left panel) and EFV (right panel) with observed average profile. Red dashed line: a threshold of 200. Blue dashed line: a threshold of 350.

Fig 5 shows the estimated mean profiles for the second order FP models for patients who initiated with NVP (left panel) and EFV (right panel) treatment regimen. The increase in log(CD4) cell counts from baseline was maintained until the end of follow-up period. Based on estimated FP models we can predict the log(CD4) counts for each subject using Eq (9), for subjects under NVP and EFV, respectively,

$$\begin{array}{c} \begin{array}{cc} & \hat{f}\left(t_{ij}\right)=\left(5.22+{\hat{b}}_{0i}\right)+\left(0.05+{\hat{b}}_{1i}\right)log\left(t_{ij}\right)+\left(0.08+{\hat{b}}_{2i}\right)t_{ij}^{0.5},\\ & \hat{f}\left(t_{ij}\right)=\left(5.05+{\hat{b}}_{0i}\right)+\left(0.14+{\hat{b}}_{1i}\right)log\left(t_{ij}\right)+\left(0.024+{\hat{b}}_{2i}\right){\left(log\left(t_{ij}\right)\right)}^{2}, \end{array} \end{array}$$ (9)

Here, ${\hat{b}}_{0i}$, ${\hat{b}}_{1i}$ and ${\hat{b}}_{2i}$ are the empirical Bayes estimates for the subject specific random effects. The fixed effect estimates are given in S1 Tables.

**Fig 6:**
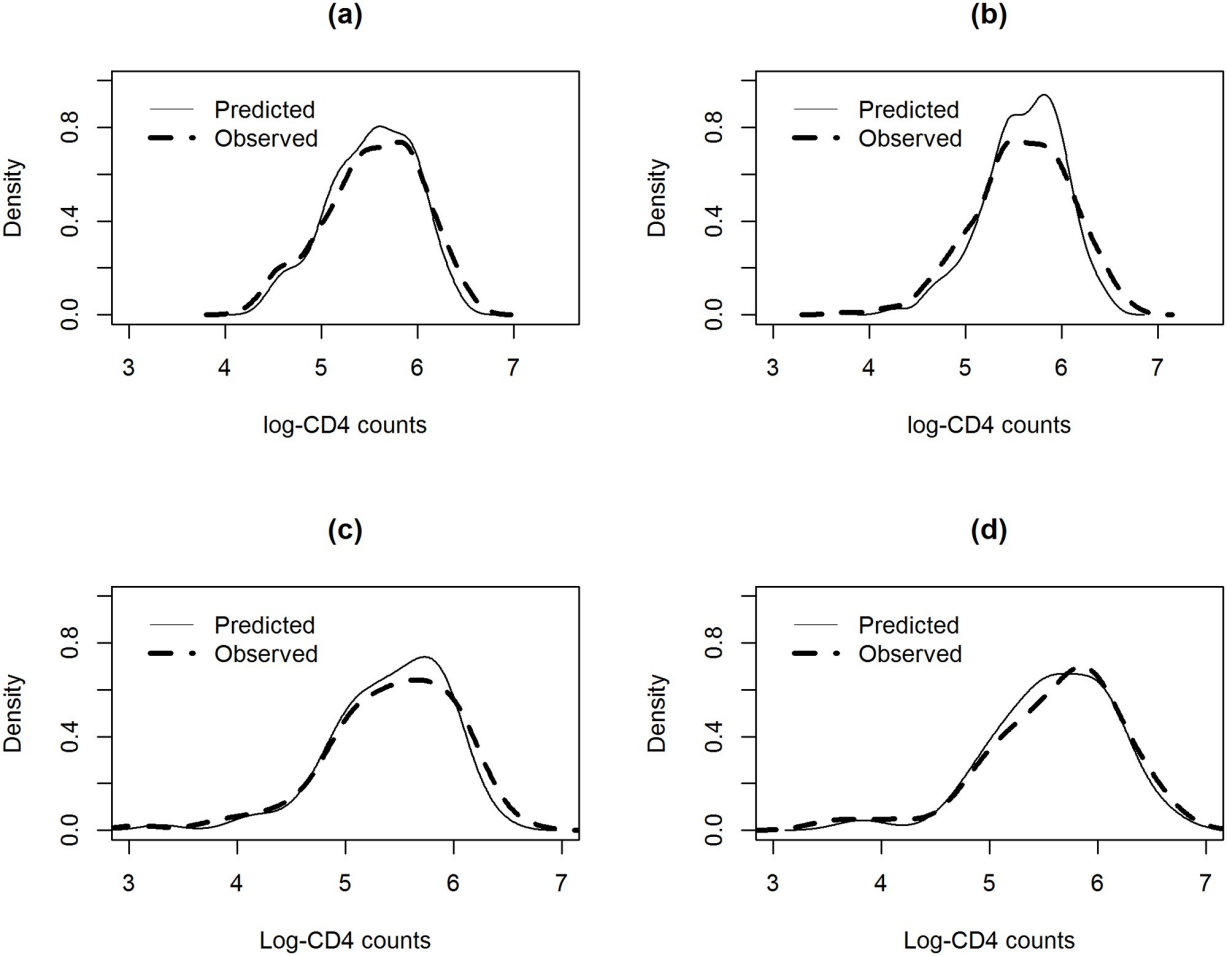
Density estimates for the distribution of the observed values (dashed line) and model-based estimation (solid line) Panel a: NVP, 6 months. Panel b: NVP: 12 months. Panel c: EFV, 6 months. Panel d: EFV: 12 months.

The density estimate for the distribution of the estimated and observed values for NVP and EFV at 6 and 12 months are shown in Fig 6 and indicates that the model is performing well in terms of Estimation at time points within the estimation period.

**Fig 7:**
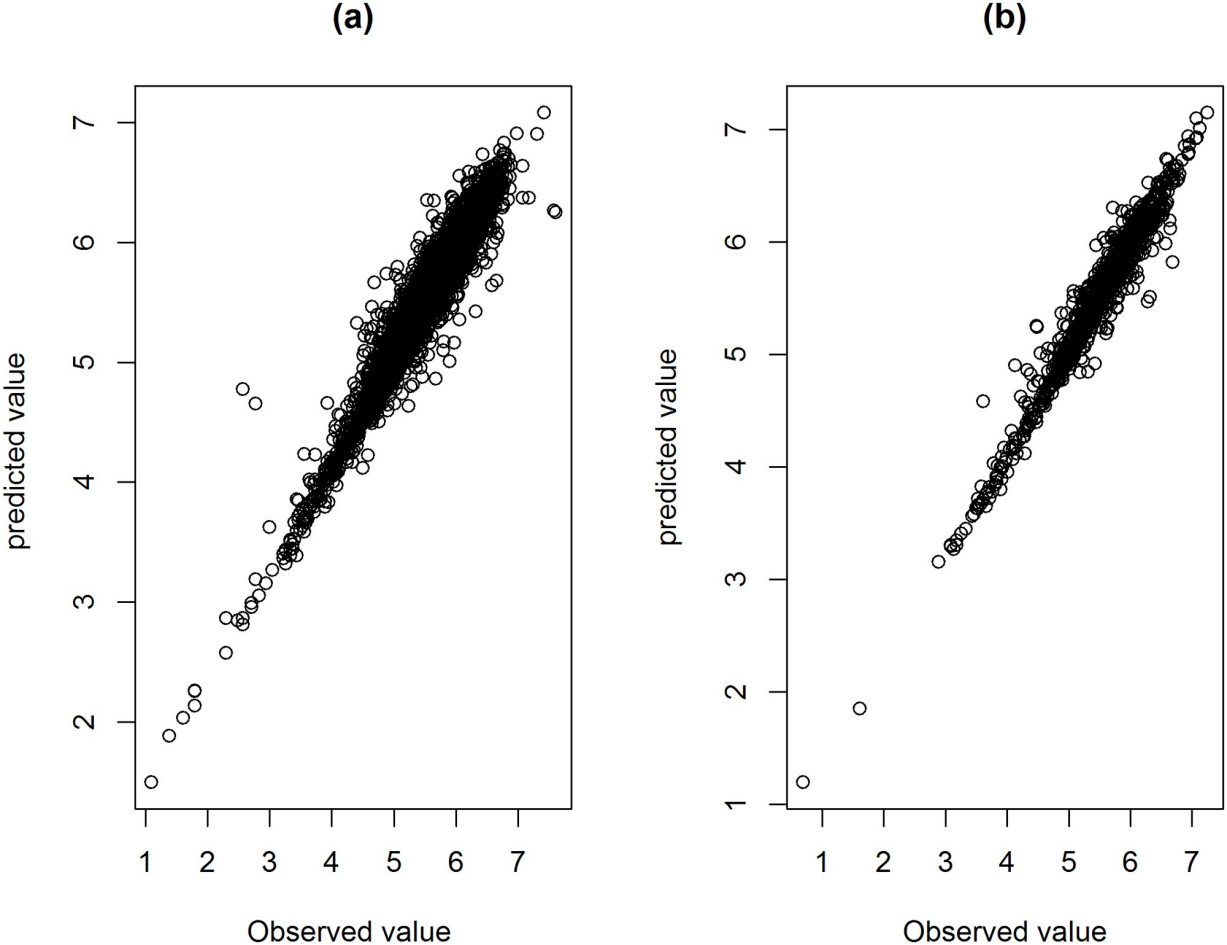
Observed versus model based predicted values for 0-68 months. Panel a: NVP. Panel b: EFV. Note that all data are used to estimate the predictive model.

Observed and predicted values at all time points are presented in Fig 7 which reveals a strong positive correlation between the estimated and observed values (0.966 and 0.977 for NVP and EFV, respectively).

### Model based prediction of CD4 cell counts: Estimation period 0-30 months

**Fig 8:**
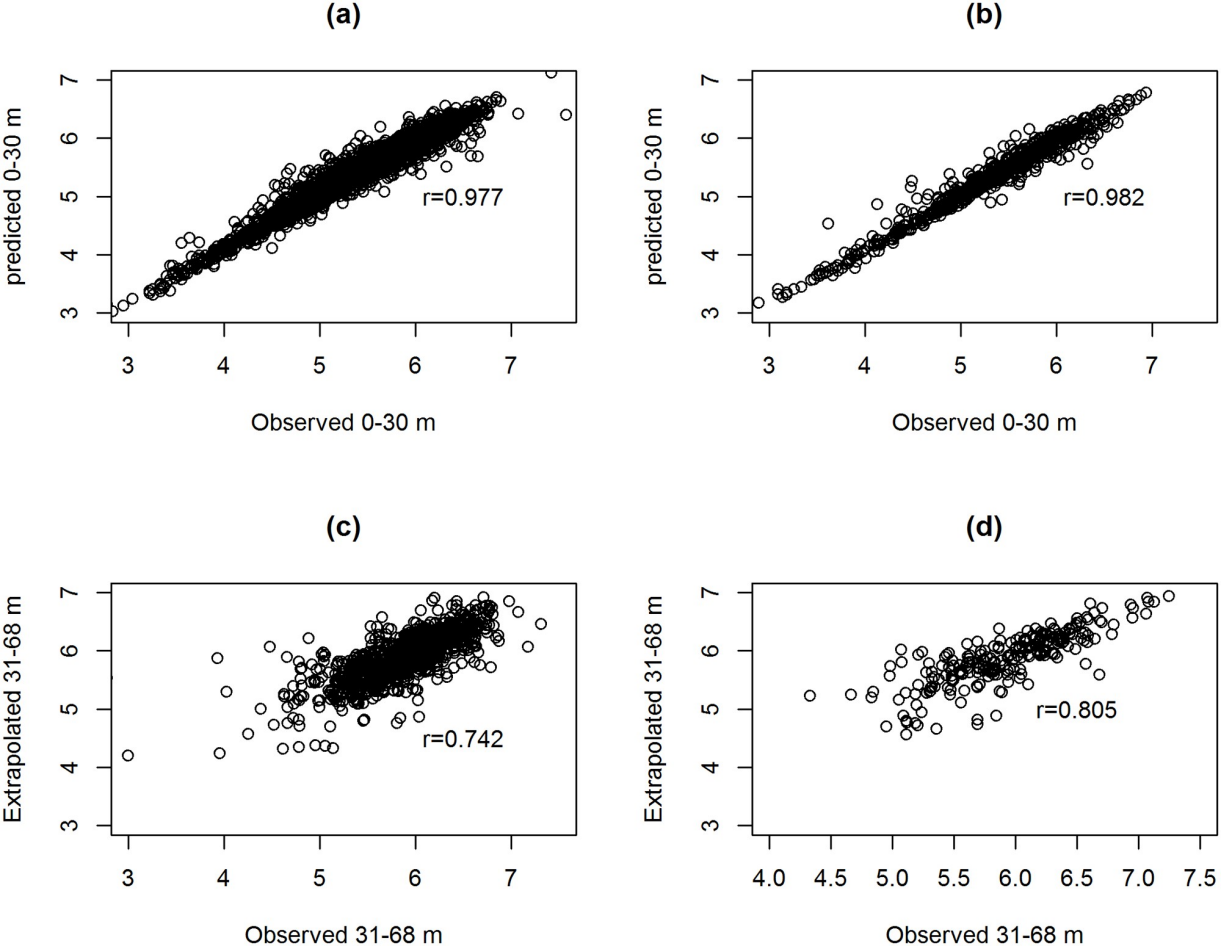
Observed versus model based predicted of CD4 counts obtained from a model that was estimated within the estimation period (0-30 months). Panel a and b: observed versus estimated values in the estimation period for NVP and EFV, respectively; Panel c and d are observed versus predicted values in the prediction period for NVP and EFV, respectively.

In the previous section model based prediction were obtained using FP2 models which were estimated using all data. In this section the data is divided into two periods. The first, 0-30 months, is used for the estimation of the model parameters while the second, 31 to 68 months, is used for prediction. Results obtained from a sensitivity analysis with different length for the estimation and the prediction period are given in the supplementary appendix of the manuscript (S1 Appendix). The analysis was performed for estimation periods of 0-24 months and 0-36 months. Fig 8a and 8b present the observed and predicted values of log(CD4) counts within the estimation period and reveal, similar to the previous section, high positive correlation (0.976 for NVP and 0.982 for EFV). Fig 8c and 8d display the predicted versus observed log(CD4) counts within the prediction period. Note that for this period the data were not used for the estimation of the model parameters. As expected, the correlations decrease to 0.805 and 0.742 for EFV and NVP, respectively.

**Fig 9:**
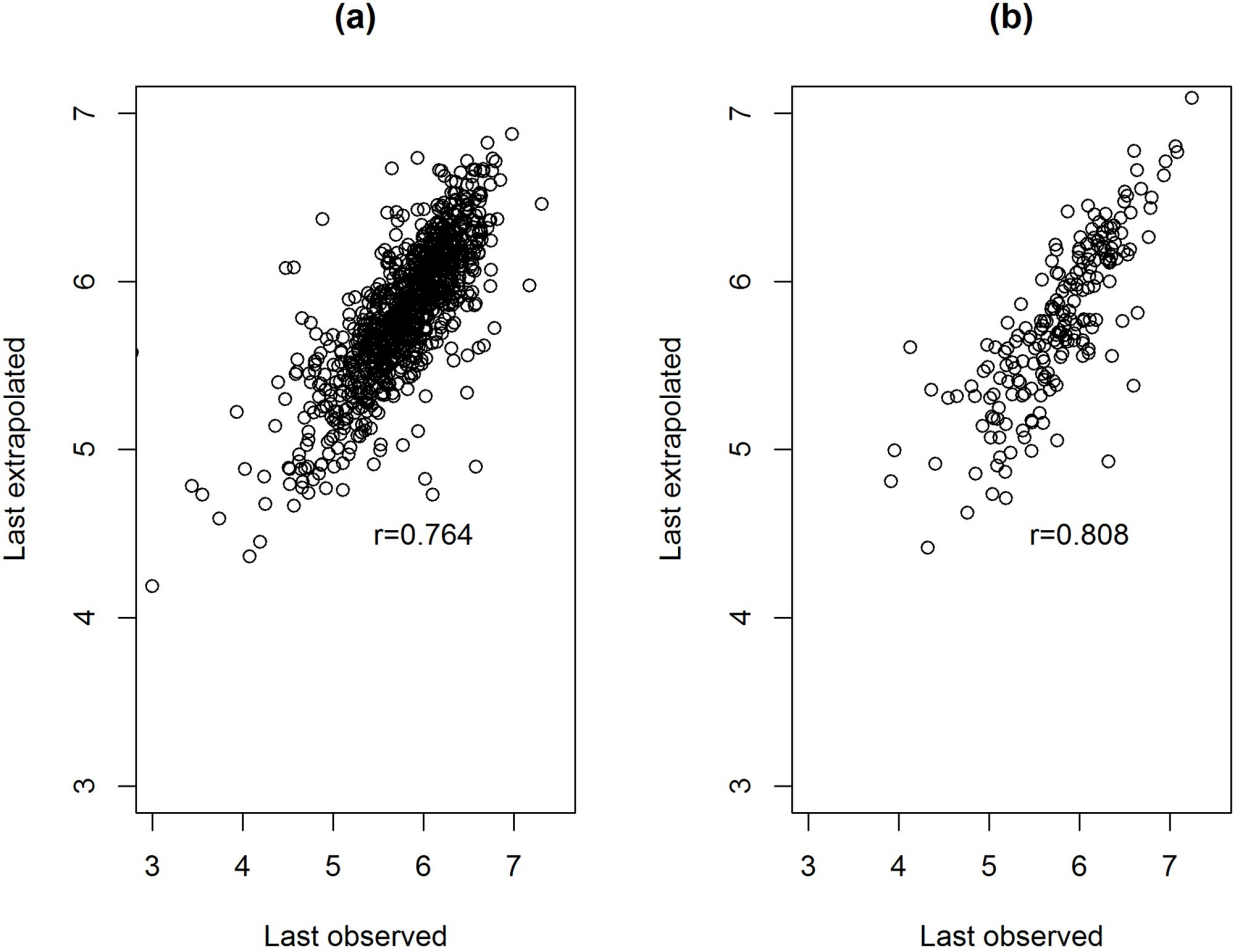
Last observed CD4 count and model based prediction based on a model which was estimated within the estimation period. Panel a: Patients who initiated with NVP containing regimen. Panel b: patients who initiated with EFV containing regimen.

The FP model estimated within the estimation period can be used to predict a subject specific last observed log(CD4) counts. This implies that for each subject, information about log(CD4) counts from the first 30 months of the treatment is used to predict the last observed log(CD4) count of the subject. Fig 9a and 9b present the observed and the predicted values. The correlations are equal to 0.764 and 0.808 for NVP and EFV, respectively.

### Subject specific prediction of time to cross a pre specified CD4 threshold

The FP model was used to estimate a subject specific time to cross a pre specified CD4 threshold. Three different thresholds were considered (200, 350 and 500) to estimate a model-based time to cross the threshold which was consider as an event. Note that model based predictions are obtained using a model for which the parameter were estimated using data from the estimation period (i.e., 0-30 months).

**Fig 10:**
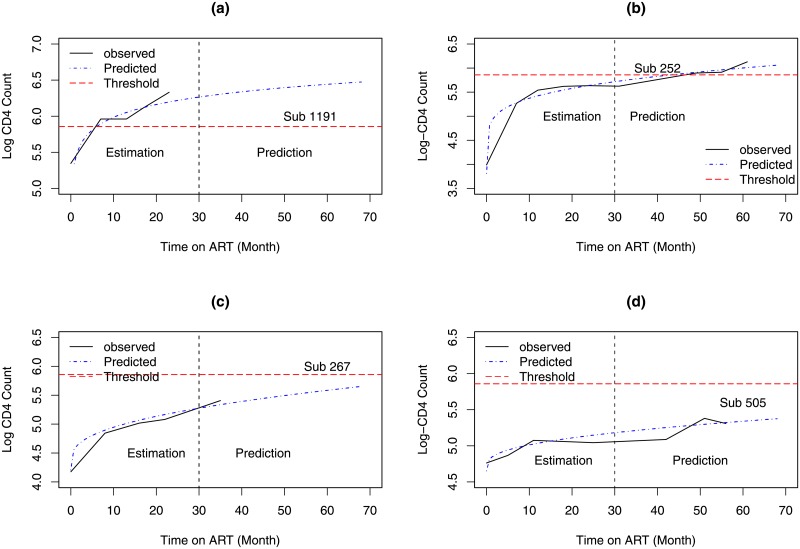
Observed and model based predicted CD4 counts for selected patients. Panel A: a patient for whom both observed and predicted values cross the threshold within the estimation period. Panel B: a patient for whom both observed and predicted values crossed the threshold after the estimation period. Panel C: a patient for whom the observed values are below the threshold and the predicted time to cross the threshold is estimated to be 74.55 months. Panel D: a patient for whom both observed and predicted values are below the threshold (350 *cells*/*mm*^3^) during the study period.

Fig 10 shows the observed and predicted log(CD4) counts for four selected patients. Panel *a* shows an example of a patient for which both the observed and predicted values cross the threshold within the estimation period. The time to cross the threshold is estimated to be 5.62 months. For patient presented in panel *b*, both the observed and predicted values cross the threshold within the prediction period (i.e 31-68 months). Panel *c* presents a patient for which the observed log(CD4) count are below the threshold of 350 and the predicted time to cross the threshold is estimated to be 74.55 months. Panel *d* show an example of patients for which both the observed and predicted log(CD4) counts remain below the threshold of 350 until the end of the follow up period.

**Fig 11:**
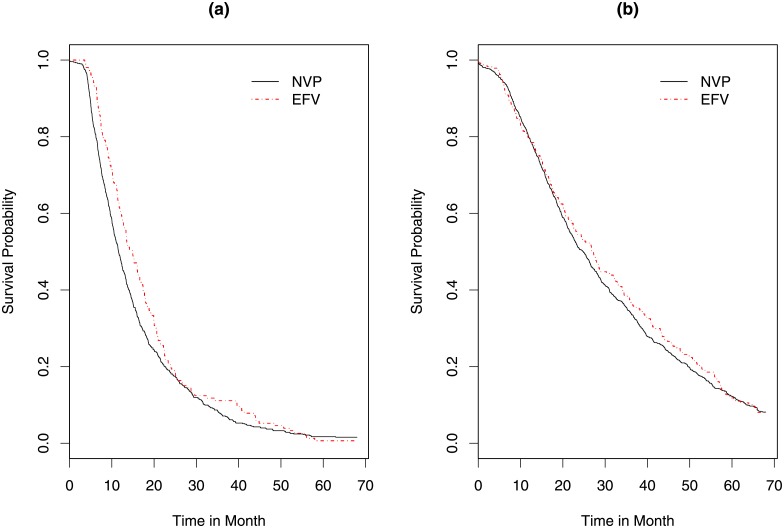
**Kaplan-Meier curve for the time to cross a pre specified threshold of CD4** *cells*/*mm*^3^ **be treatment group**; Panel a: K-M curves for threshold of 200 *cells*/*mm*^3^ for EFV and NVP containing regimen. Panel b: K-M courses for a threshold of 350 *cells*/*mm*^3^ for EFV and NVP containing regimen.

Fig 11 presents the kaplan-Meier curves for the time to cross the threshold of 200 and 350 *cells*/*mm*^3^. For the threshold of 200 CD4 *cells*/*mm*^3^, there is a significant difference between EFV and NVP groups (p values for the log rank test is 0.0422). The median time to cross the threshold is estimated to be equal to 11.6 months (95% CI: 10.8-12.4) for NVP group and 15.0 months (95% CI: 12.7-17.3) for the EFV group. When the threshold increases to 350 (see Fig 11, panel *b*), the distribution of the time to cross the threshold of the groups were comparable (p-value = 0.52).

**Fig 12:**
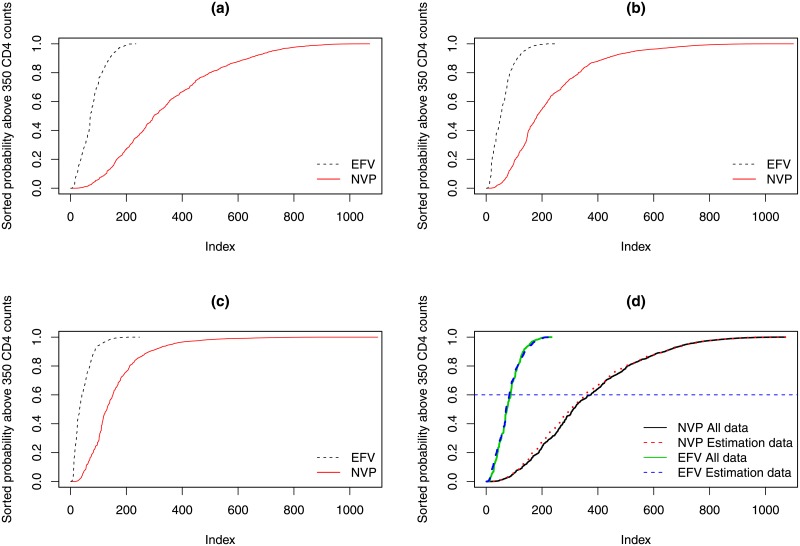
Sorted probabilities to cross a threshold of 350 CD4 counts. Panel a: sorted probability to cross the threshold at 60 months. Panel b: sorted probability to cross the threshold at 90 months. Panel c: sorted probability to cross the threshold at 120 months. Panel d: Sorted probability at 60 months obtained for models which were estimated within the estimation period and when using all data.

The predicted probabilities to cross a threshold of 350 *cells*/*mm*^3^ at 60, 90 and 120 months were calculated according to Eq 3 and shown in Fig 12. For all time points, the probability to cross a threshold of 350 *cells*/*mm*^3^ is higher for the EFV group. Although, the difference between the two treatment groups is higher for lower months, in the long run the two treatment groups had comparable result.

For example, at 60 months, the probability to cross a threshold of 350 *cells*/*mm*^3^ higher than 0.5 was calculated for 160 patients who were initiated on EFV (65% from the EFV sample) compare to 760 patients who were initiated with NVP (69% of the NVP sample). Fig 12d shows sorted predicted probabilities calculated using two models: (1) the black solid and green dash lines represent sorted probability for NVP and EFV regimens when the model is fitted for all available data, respectively and (2) the red and blue dash lines represent sorted probability for NVP and EFV regimens when the model is within the estimation period, respectively. We note that the two models lead to comparable probabilities in the estimation period and all observed dataset.

The procedures described in the methods section allows us to use Eq 3 to estimate both the time and the probability to be above a given threshold. For illustration we use 8 patients. Patients 2077, 1191, 67 and 783 are initiated with EFV containing regimen, while patients 44, 240, 747, and 252 are initiated with NVP regimen were selected. Table 1 shows the estimated time to cross the threshold and the corresponding 95% confidence interval for the selected patients.

**Table 1:** Estimated time to cross the threshold and 95% confidence interval for selected individuals.

PatientID	Baseline CD4	≥ 200*cells*/*mm*^3^		≥ 350*cells*/*mm*^3^		≥ 500*cells*/*mm*^3^	
		${\hat{⊤}}_{\tau }$	95%CI	${\hat{⊤}}_{\tau }$	95%CI	${\hat{⊤}}_{\tau }$	95%CI
**EFV**							
2077	425	2.8x10^−3^	(3.7x10^−4^-1.1510^−2^)	1.376	(1.045-1.763)	10.312	(9.897-10.890)
1191	210	2.789	(2.392-2.981)	9.752	(9.456-10.081)	19.786	(19.336-20.486)
67	126	13.667	(12.737-14.495)	42.918	(37.654-52.951)	79.928	(55.524-82.962)
783	67	20.282	(19.189-21.052)	51.361	(48.968-54.427)	82.1640	(71.872-85.256)
**NVP**							
44	490	2.5x10^−3^	(5.4X10^−4^, 9.0x10^−3^)	1.601	(1.177-2.142)	16.717	(16.023-17.624)
240	271	1.406	(0.975-1.501)	12.102	(11.954-12.259)	21.481	(21.001-22.085)
747	138	6.711	(6.765-7.096)	28.107	(27.259-29.968)	69.545	62.335-72.333
252	54	10.728	(10.473-11.027)	39.161	(38.496-40.721)	69.422	(67.492-71.566)

Patients 783 and 252 started ART when their CD4 counts dropped lower than 100 *cells*/*mm*^3^. The estimated time to cross 200 *cells*/*mm*^3^ threshold for these patients were 20.282 (95%CI: 19.189-21.052) and 10.728 (95%CI:10.473-11.027), respectively. Patients 67 and 747 were initiated ART at CD4 counts between 100 and 200 *cells*/*mm*^3^. These patients are expected to reach 200 *cells*/*mm*^3^ threshold at 13.667 (95%CI:12.737-14.495) and 6.711 (95%CI: 6.765, 7.096) months, respectively.

**Fig 13:**
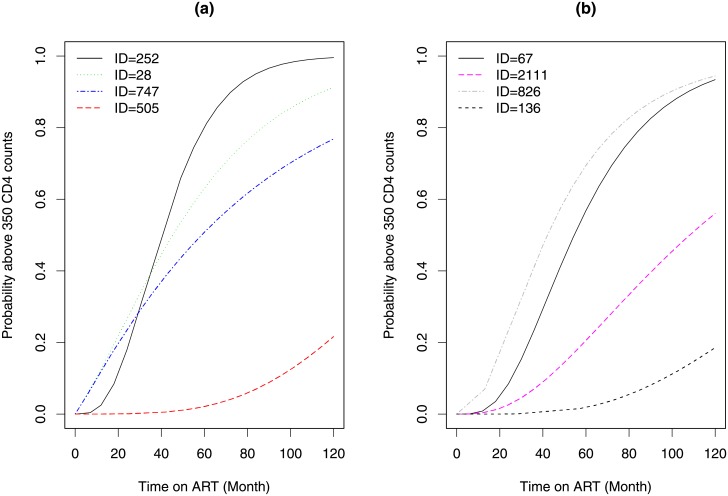
Probabilities to cross a threshold of 350 *cells*/*mm*^3^ for selected subjects. Panel a is for patients who initiated with NVP containing regimen, and panel b is for patients who initiated with EFV containing regimen.

Patients 1191 and 240 have CD4 count greater than 200 *cells*/*mm*^3^ when they start ART. The expected time to cross threshold 350 *cells*/*mm*^3^ were 9.752 (95%CI: 9.456-10.081) and 12.102 (95%CI: 11.954-12.259), respectively. Patients 2077 and 44 were initiated ART at CD4 cell counts greater than 350 *cells*/*mm*^3^. The estimated times to reach the threshold 500 *cells*/*mm*^3^ were 10.312 (95%CI: 9.897-10.890) and 16.717 (95%CI: 16.023-17.624), respectively. For each patient, the probability to cross the threshold of 350 *cells*/*mm*^3^, a different time points, was calculated according to Eq 5 and presented in Fig 13.

## Discussion

In this paper we have shown that model based predictions are highly correlated with the observed values within the estimation period. Considerable relationship was observed in the prediction period as well. This provide evidences that the proposed model can be used for long-term prediction of unobserved CD4 cell counts. The density estimates for the distribution of observed and predicted values supported these relations. Other studies used similar approach for long term prediction [32, 33]. The mixed effects FP model allows us to estimate the distribution of the time to cross a pre specified CD4 cell count threshold of interest and to use this distribution to compare between treatments. More than half (52.87%) of the patients who initiated ART at CD4 cell counts less than 200 *cells*/*mm*^3^ cross the threshold in six months period after initiation. When the threshold is 350 *cells*/*mm*^3^, the proportion who crossed the threshold at 6, 24, 36 and 48 months were 13.88%, 40.14%, 52.79% and 61.65%, respectively. Several observational studies have reported that the probability of attaining elevated CD4 level can be sustained for at least 7 years and probably indefinitely [15, 34–36].

Patients who initiated at higher CD4 count get more pronounced CD4 cell count rise quickly than those who initiated at lower CD4 cells counts. Different studies showed that the baseline CD4 cell count influences the rate of immune reconstitution [34, 37–39]. These studies indicated that CD4 count at the time of ART initiation are critical determinants of the progression while under ART. This might be due to the fact that when the immune system is damaged, the risk of illness will increase.

We have shown that model based predictions of the time to cross a threshold reveal the similar patterns. There is difference in the time taken to cross the threshold among the two art regimens. The expected time to reach lower threshold is shorter for patients who initiated with NVP than EFV containing regimen. Whilst the expected time to reach higher threshold (350 *cells*/*mm*^3^) is shorter for EFV containing regimen which is also supported by other studies [40, 41]. This might be due to the high potency nature of EFV containing regiment.

The Kaplaan-Meier survival curve also shows that the median time to cross the thresholds 200 CD4 *cells*/*mm*^3^ was shorter for patients who had been initiated with NVP as compared to EFV. Similar trend was reported by other studies [41, 42]. The possible reason is NVP has been used for patients with low CD4 level to reduce the side effect of EFV.

The limitation of this study was the unmeasured variables effect on the findings of the study which includes income, occupation, nutrition status and viral load. Note that the models presented in this manuscript included a limited number of covariates which were available in the database. Other covariates, if available in the database, such as TB status etc., can be included in the same way. The fractional polynomials model discussed in this paper was applied to a data based in which CD4 counts was the response variable for the assessment of patient progression under ART. In case that viral load measurements are available and used for progression assessment, the fractional polynomial modelling framework and the estimation procedure discuss in this paper can be used to model the data. Moreover, additional covariates, if available, can be included in the model as well.

## Conclusions

In conclusion, the model was used to estimate the probability of an individual to have CD4 count above a pre-specified threshold. By predicting the long-term outcomes of CD4 count of a patient one can advise patients about the potential ART benefits that accrue in the long term. Initiation of ART at higher CD4 cell counts has more benefit in achieving immunological success at a faster rate. Efavirenz containing regimen improves CD4 cells counts of the patient quicker than NVP containing regimen for higher baseline CD4 cell counts. Hence, those who have higher baseline CD4 cells count can be initiated with EFV containing regimen.

## Supporting information

S1 Fig Plots of powers for the first order fractional polynomial versus AIC value from the model. The left panel shows the plots of first degree powers against AIC for NVP and the right panel shows the plot of first degree powers against AIC for EFV.
(TIFF)

S2 Fig Plots of powers for the second order fractional polynomial versus AIC value from the model. Panel (a) shows the plots of first and second powers against AIC for NVP and Panel (b) shows the plot of first and second powers against AIC for EFV.
(TIFF)

S1 Tables Results of model comparison using akaike information criterion and model parameter estimates and their associated standard error using FP2. (PDF)

S1 Appendix Sensitivity analysis, when estimation period is 0-24 months and 0-36 months. Panel (a) and (b) show the plots of observed and predicted values in the estimation period for NVP and EFV regimens, respectively. Panel (C) and (d) show the plots of observed and predicted values in the prediction period for NVP and EFV regimens, respectively.
(PDF)

S2 Appendix De-identified minimal data. (CSV)
