# Supplementary figures and images for: Model-based prediction of CD4 cells counts in HIV-infected adults on antiretroviral therapy in Northwest Ethiopia: A flexible mixed effects approach
Source: PLoS One. 2019 Jul 10;14(7):e0218514. doi: 10.1371/journal.pone.0218514 (PMC6619674; doi:10.1371/journal.pone.0218514)

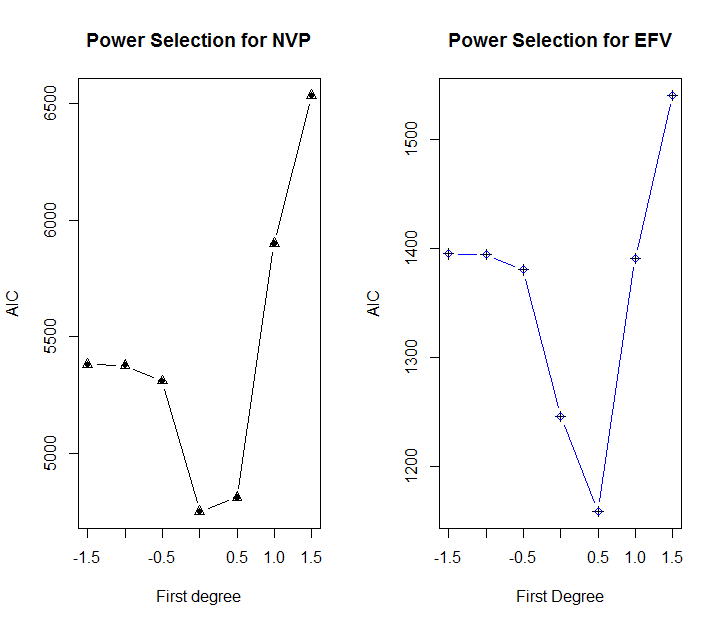

Supplement: S1 Fig — The left panel shows the plots of first degree powers against AIC for NVP and the right panel shows the plot of first degree powers against AIC for EFV. (TIFF) [file pone.0218514.s001.tiff]

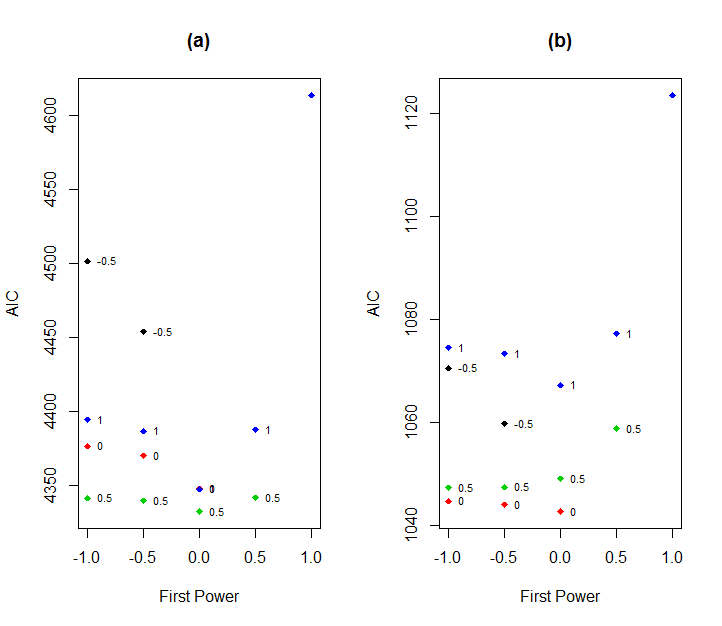

Supplement: S2 Fig — Panel (a) shows the plots of first and second powers against AIC for NVP and Panel (b) shows the plot of first and second powers against AIC for EFV. (TIFF) [file pone.0218514.s002.tiff]
