# Supplementary material for: Model-based prediction of CD4 cells counts in HIV-infected adults on antiretroviral therapy in Northwest Ethiopia: A flexible mixed effects approach
Source: PLoS One. 2019 Jul 10;14(7):e0218514. doi: 10.1371/journal.pone.0218514 (PMC6619674; doi:10.1371/journal.pone.0218514)
Supplement: S1 Tables — (PDF) [file pone.0218514.s003.pdf]

Appendix

Table 1. *Application of the Functional Selection Procedure to NVP and EFV Containing ART regimens*

Model	Power	Comparison	-LogLL	LogLL diff.	p-value
			NVP		
Null	-	Linear vs Null	8972.7	2901.1	<0.0001
Linear	1	FP1 vs Linear	6071.6	1334.4	<0.0001
FP1	0	FP21 vs FP1	4737.2	293.2	<0.0001
FP2	0 ; 0.5	FP21 vs FP22	4444.0	131.3	<0.0001
			EFV		
Null	-	Linear vs Null	1867.4	489	<0.0001
Linear	1	FP1 vs Linear	1378.4	259.9	<0.0001
FP1	0.5	FP21 vs FP1	1121.5	19.5	0.0002
FP2	0;0	FP21 vs FP22	1102.0	79.2	<0.0001

Table 1 shows that two degrees fractional polynomial mixed effect model with intercept, power 1 and 2 time as random effects (FP2) were found to be the best model for both NVP and EFV treatment group.

Table 2. *Model parameter estimates and their associated standard error using FP2. The p-values are based on the Wald test.*

Effect	Estimate	Estimate(s.e)	P-value
		NVP	
β_0	5.619	0.062	<0.0001
$log(time)$	0.033	0.003	<0.0001
$time^{0.5}$	0.085	0.006	<0.0001
Age	-0.008	0.002	<0.0001
Male	-0.119	0.032	0.0003
WHO II	-0.102	0.041	0.0129
WHO III	-0.098	0.034	0.0044
WHO IV	-0.196	0.071	0.0061
		EFV	
β_0	5.368	0.170	<0.0001
$log(time)$	0.161	0.010	<0.0001
$time^2$	0.021	0.002	<0.0001
Age	-0.002	0.004	0.573
Male	-0.179	0.069	0.011
WHO II	-0.073	0.135	0.586
WHO III	-0.267	0.103	0.010
WHO IV	-0.150	0.124	0.227
