# Supplementary material for: Model-based prediction of CD4 cells counts in HIV-infected adults on antiretroviral therapy in Northwest Ethiopia: A flexible mixed effects approach
Source: PLoS One. 2019 Jul 10;14(7):e0218514. doi: 10.1371/journal.pone.0218514 (PMC6619674; doi:10.1371/journal.pone.0218514)
Supplement: S1 Appendix — Panel (a) and (b) show the plots of observed and predicted values in the estimation period for NVP and EFV regimens, respectively. Panel (C) and (d) show the plots of observed and predicted values in the prediction period for NVP and EFV regimens, respectively. (PDF) [file pone.0218514.s004.pdf]

Model-based Prediction of CD4 Cells Counts in HIV-Infected Adults on Antiretroviral Therapy in Northwest Ethiopia: A Flexible Mixed Effects Approach

Supplementary appendix

Tadesse Awoke Ayel et al.

Sensitivity analysis for the length of the estimation period

As mentioned in the paper, the total length of follow up period is 68 months. For the analysis presented in the paper, the estimation period is 30 months. In this supplementary appendix, we present a sensitivity analysis In which two estimation periods were considered; (1) 0-24 months and (2) 0-36 months.

Similar to the analysis presented in the paper, for the first senario, a length of 0-24 months was used to build the model and prediction was for the period of 25-68 months (the prediction period). The model was used to predict the CD4 counts values for NVP and EFV containing regimen. The correlation between observed and predicted values, within the estimation period (0-24 months) were found to be 0.9788 and 0.9832 for NVP and EFV containing regimens, respectively (see panel a and b in Figure 1). Based on the model build in the estimation period, prediction was made for the period of 25-68 months. The correlations between the observed and predicted values in the prediction period were 0.7272 and 0.7589 for NVP and EFV, respectively (see panel c and d Figure 1).

A second analysis was performed for an estimation period of 0-36 months. The correlations between observed and predicted values in the estimation period were 0.9722 and 0.9809 for NVP and EFV containing regimen, respectively (see panel a and b in Figure 2). The correlation

between observed and predicted values in the prediction period (37-68 months) were 0.8346 and 0.8074 for NVP and EFV, respectively (see panel c and d Figure 2).

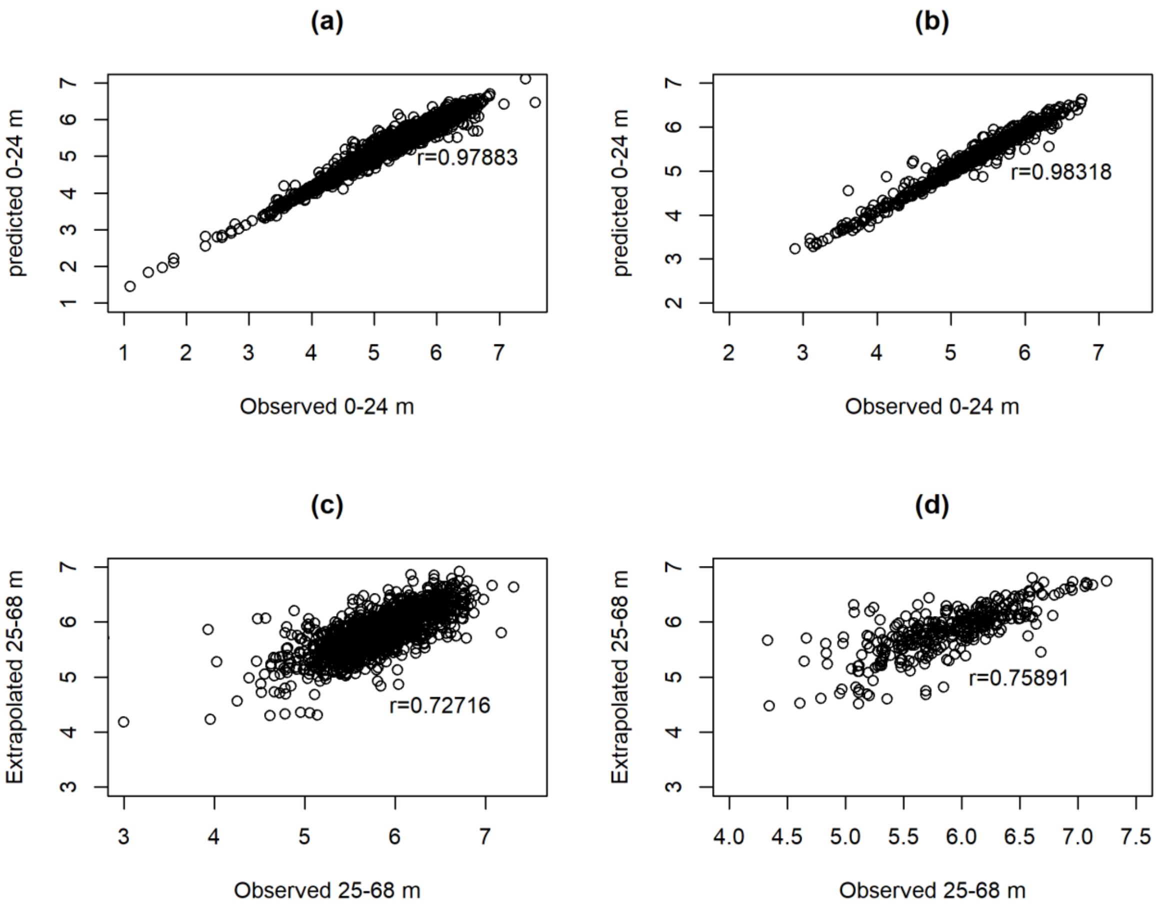

Figure 1: Sensitivity Analysis, when estimation period is 0-24 months. Panel (a) and (b) show the plots of observed and predicted values in the estimation period for NVP and EFV regimens, respectively. Panel (C) and (d) show the plots of observed and predicted values in the prediction period for NVP and EFV regimens, respectively.

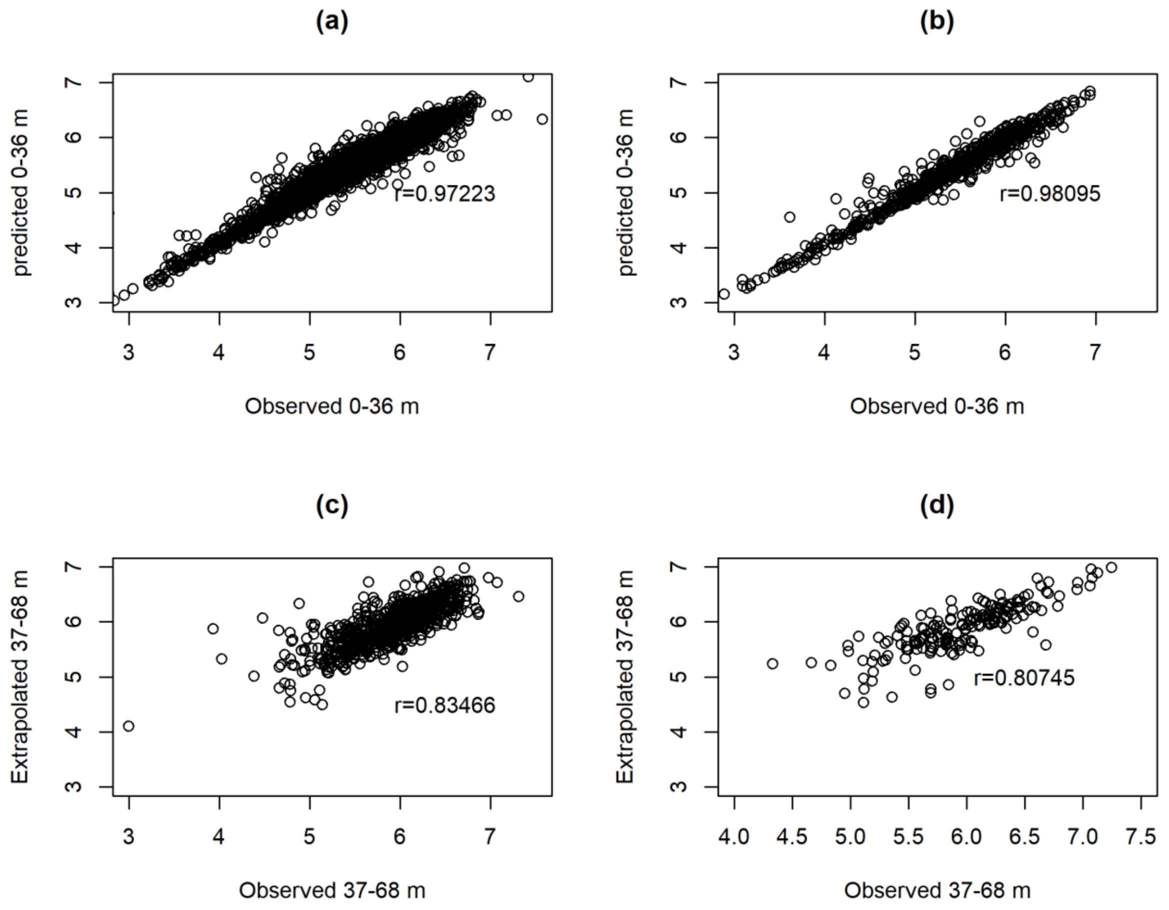

Figure 2: Sensitivity Analysis, when estimation period is 0-36 months. Panel (a) and (b) show the plots of observed and predicted values in the estimation period for NVP and EFV regimens, respectively. Panel (C) and (d) show the plots of observed and predicted values in the prediction period for NVP and EFV regimens, respectively.

Propensity score matching

The R function “matchit” was used the nearest neighbour matching method to compute the PS and the resulting plots are in Figure 1 and Figure 2.

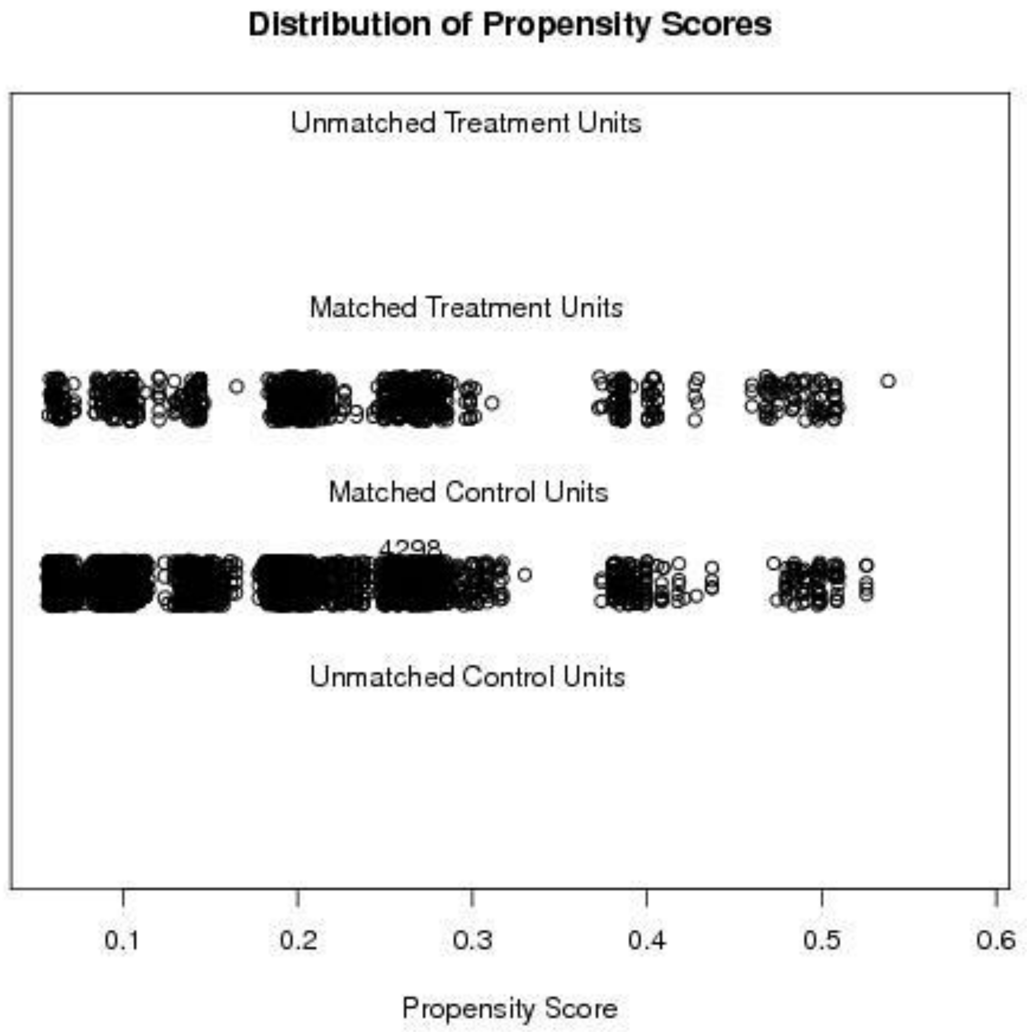

Figure 3: Distribution of propensity scores after and before matching.

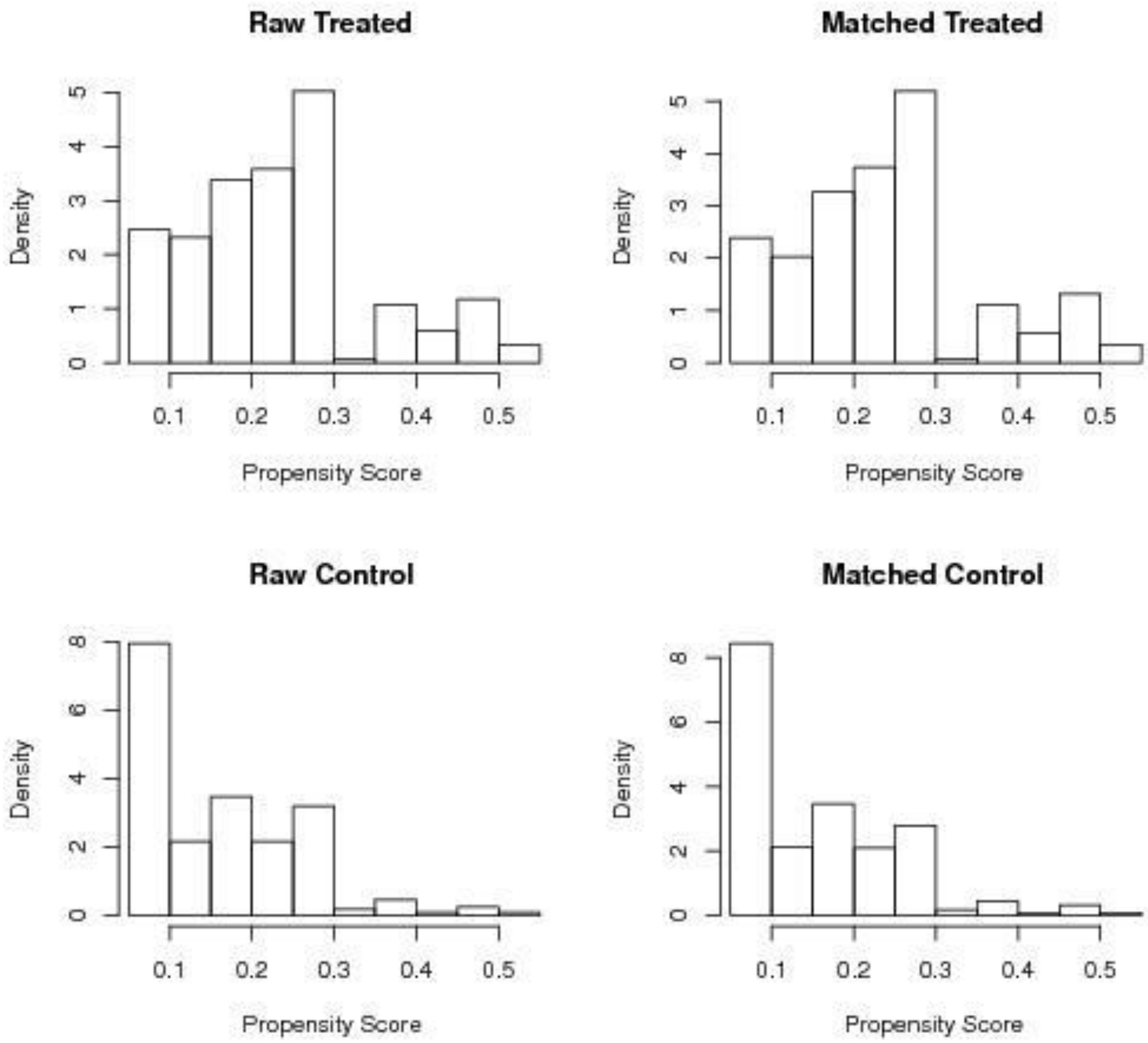

Figure 4: Histogram for the row and matched groups.
